# The relationships among Taiwanese youth’s polychronicity, multitasking behavior and perceived learning performance in online learning

**DOI:** 10.3389/fpsyg.2023.1131765

**Published:** 2023-04-12

**Authors:** Yi Fang Luo, Seokmin Kang, Shu Ching Yang, Chia Mei Lu

**Affiliations:** ^1^Graduate School of Human Sexuality, Shu-Te University, Kaohsiung, Kaohsiung County, Taiwan; ^2^Department of Teaching and Learning, College of Education & P-16 Integration, The University of Texas Rio Grande Valley, Edinburg, TX, United States; ^3^Institute of Education, National Sun Yat-sen University, Kaohsiung, Taiwan; ^4^Department of Digital Multimedia Design, Cheng Shiu University, Kaohsiung, Taiwan

**Keywords:** polychronicity, multitasking, learning performance, online learning, knowledge acquisition, learning satisfaction

## Abstract

**Background:**

The advancement of digital technology implies the importance of polychronic learning. Since polychronicity is not equivalent to multitasking behavior, they need to be considered separately. However, less research has been explored on how polychronicity is related to multitasking behavior in the educational field.

**Objective:**

To explore the relationships among polychronicity, multitasking behavior and learning performance (including knowledge acquisition and learning satisfaction) in an online learning environment.

**Methods:**

The relationship among variables was analyzed from 865 responses obtained from a questionnaire survey, and independent sample *t* tests and SEM analysis were used to examine the research hypotheses.

**Results:**

College students showed a higher frequency of multitasking behavior, time tangibility and scheduling preference, and learning satisfaction in multitasking online learning environments than high school students. Additionally, college students were different from high school students on the paths of involvement with people to multitasking behavior (Δ *χ*^2^= 5.42, *p* = 0.02) and scheduling preference to learning satisfaction (Δ*χ*^2^ = 9.54, *p* = 0.002).

**Conclusion:**

The relationship among polychronicity, multitasking behavior and perceived learning performance in an online learning environment varies by student educational stage.

## 1. Introduction

COVID-19 has forced educational institutions to promptly respond to this emergency and take a big step forward from traditional face-to-face learning to online learning to ensure that education continues (Gumede and Badriparsad, 2022). Previous studies have pointed out the advantages of online learning, including time and cost effectiveness (Elida et al., 2012; Almahasees et al., 2021), flexible use of time and space (Lemay et al., 2021; Maqableh and Alia, 2021; Turan et al., 2022), and the repetitive learning features of online resources (Bdair, 2021). These online resources are considered especially important for students’ self-directed learning, but research also points out that students’ online self-directed learning is challenging because students report that they are easily distracted and have limited attention (Lemay et al., 2021; Maqableh and Alia, 2021), which in turn affects their academic performance (Kim et al., 2022).

Distraction and inattention may be exacerbated by learners’ multitasking behavior (MB; Cebollero-Salinas et al., 2022). However, time-limited multitasking seems to be different from the current online learning ethos that emphasizes flexibility and self-direction. The recent education reform in Taiwan requires the cultivation of interdisciplinary, independent, and autonomous talent, prompting self-directed online learning to become the focus of education in Taiwan and emphasizing students’ personalized learning processes rather than learning within a given time (Chen and Li, 2021). Given the lack of research on MB in online learning, one of the study’s purposes is to explore MBs in online learning and students’ perceived learning performance.

Multitasking behavior is closely related to polychronicity. Polychronicity is an individual’s natural tendency or preference for constructing time (Sanderson, 2012; Capdeferro et al., 2014), specifically defined as an individual’s preference to participate in two or more tasks or events simultaneously and the belief that one’s preference is the best way to do things (Bluedorn et al., 1999). Although studies of polychronicity are rare in educational settings, previous studies have shown differences in multitasking behavior among students at different levels of education (e.g., Jeong et al., 2010). However, polychronicity needs to be discussed more in online education. Based on the relationship between multitasking behavior and learning outcomes, as well as the relationship between multitasking behavior and polychronicity, this study explores the relationship between polychronicity, multitasking behavior, and learning outcomes by considering differences in educational stages.

## 2. Literature review

### 2.1. Online multitasking and learning performance

Distraction and inattention are often seen as a result of a learner’s multitasking behavior (Cebollero-Salinas et al., 2022). Learning multitasking behavior is defined as distraction and nonsequential task switching of ambiguous tasks performed in a learning environment (Chen and Yan, 2016), and multitasking behavior seems to be more likely to arise in an online learning environment because information technology (IT) combined with the internet has brought changes to the working patterns in time allocation (Lee and Perry, 2001). IT makes learning no longer limited by time and space, thus changing traditional classroom experiences to being intermittent, multidirectional, and nonsequential and easily disrupting the traditional view of time and space in learning activities (Capdeferro et al., 2014). This also means that IT provides the opportunity for learners to multitask. Sun and Zhong (2020) pointed out that media users are more likely to engage in multitasking behavior and behavioral responses to new information and communication technology applications in today’s mobile media era. Media multitasking behavior can be defined as using more than two types of media at the same time (Rideout et al., 2010) or quickly switching between tasks on the same media, such as working with multiple browsers or using several types of software simultaneously (Cardoso-Leite et al., 2015).

Multitasking behavior is often used to explore student learning outcomes. However, previous research has been administered mainly in the context of multitasking learning in a given time period to find the relationship between learning outcomes and multitasking behaviors, revealing that multitasking learning behaviors lead to anxiety (e.g., Seddon et al., 2021), poorer academic performance (e.g., Loh et al., 2016), or perceived lower learning performance (e.g., Fried, 2008).

### 2.2. Online multitasking and polychronicity

Szumowska et al. (2018) noted the difference between behavior and preference and argued that multitasking behavioral performance does not represent an individual’s preference for multitasking. Preference for multitasking is nearly equivalent to polychronicity (Szumowska et al., 2018). As a natural trend or preference for constructing time (Sanderson, 2012; Capdeferro et al., 2014), polychronicity is defined as the preference for simultaneous involvement in two or more tasks or events and the belief that one’s preference is the best way to do things (Bluedorn et al., 1999). This definition of general polychronicity was adapted to the computing context and called computer polychronicity (Davis et al., 2009).

According to Palmer and Schoorman (1999), there are three distinct dimensions that are typically associated with polychronic structure: time-use preference, context, and time tangibility. These three dimensions were again applied to individual-level polychronicity and called scheduling preference (SP), involvement with people (IP) and time tangibility (TT; Capdeferro et al., 2014). Polychrons refer to those who have polychronic character. First, they value interpersonal interaction and involvement with other people (high IP). Therefore, they cross the boundaries between work and nonwork domains, such as social and leisure activities. Second, polychrons favor simultaneous activities (high SP). They prefer doing many things at the same time, show tolerance to multitasks at a given time, and effectively address interruptions and unpredictability. Finally, polychrons have time-use preferences and believe that time is not tangible and cannot be managed but is the background for an event. Since time is not a tangible resource for them, people in high-context cultures do not think they have to finish their work in a certain period of time or think that it is problematic to leave tasks unfinished (Hecht and Allen, 2005; Capdeferro et al., 2014). However, Luo et al. (2021) found that polychrons in an IT-supported learning environment prefer to work in a timely, time-saving and schedule-based manner (high TT). As polychrons are more likely to undertake multiple tasks, they may be able to achieve more goals than monochrons under the same work conditions. Thus, time management enables polychrons to complete tasks on time and even complete more tasks. Given this, this study proposes the following hypothesis:

*H*_1_: Students tend to be more polychromic (including time tangibility, involvement with people, and scheduling preference) and their multitasking behavior is more frequent.

### 2.3. Polychronicity and learning performance

Capdeferro et al. (2014) argued that we may better understand students’ learning behaviors in IT environments with the concept of polychronicity because various learning activities under IT environments occur in a polychronic manner, such as web browsing, interacting with peers on discussion boards, and responding to a teacher. Therefore, compared to monochronic learners, polychronic learners may feel more comfortable with a more flexible timeframe and more interactive environments. This can be explained by the person–environment fit (P–E fit) theory based on the interactionist theory of behavior (Chuang et al., 2016). The P–E fit theory argues that individuals’ unique behaviors must be understood in the specific situations in which they occur (Payne et al., 1982). Interactionism regulates the interaction between personal characteristics and situations and argues that only when situational cues related to individual characteristics exist, that is, situation-trait relevance, can these individual-related characteristics produce behaviors consistent with the characteristics (Geukes et al., 2012). Based on this assumption, interactionists believes that certain environmental conditions can be adapted to individual characteristics, which can lead to better individual performance and higher satisfaction (Chuang et al., 2016). The existing research has also shown that matching personal characteristics with environmental characteristics is an effective predictor of overall work satisfaction (Hardin and Donaldson, 2014).

*H*_2_: Students tend to be more polychronic (including time tangibility, involvement with people, and scheduling preference) and their perceived learning performance is better.

Predictably, polychronicity is the most important indicator of MB and leads to such behavior (König et al., 2010; Kirchberg et al., 2015; Lepp et al., 2019). For example, Lepp et al. (2019) found that polychronicity is positively associated with multitasking during online learning activities. Polychrons may be more willing to challenge multiple tasks in a given time period and are satisfied with work that requires multitasking (Sanderson, 2012), and vice versa. Madjar and Oldham’s (2006) research indicates that polychrons experience higher time pressure in a work environment where tasks are completed sequentially than in a work environment where tasks are alternated; for monochrons, the opposite is true. However, existing polychronicity studies have mainly focused on the concept of time orientation in the workplace (Manrai and Manrai, 1995; Hecht and Allen, 2005; Kirchberg et al., 2015; Bhattacharyya et al., 2018). The time factor in IT integration has been largely ignored in learning research, and polychronicity studies are rarely found in educational settings, although learners’ time orientation is an extremely important variable in the educational environment, especially in an online learning environment, which has great potential for polychronicity (Barbera et al., 2012; Capdeferro et al., 2014). The learning process involved in IT makes events and tasks increasingly occur in a polychronic manner, and students are usually expected to engage in their learning activities accordingly (Lee and Perry, 2001; Capdeferro et al., 2014). However, not every learner feels comfortable engaging in this IT learning environment because of the differences in learners’ time perceptions (for example, polychronicity and monochronicity; Capdeferro et al., 2014). Given this, this study proposes the following hypothesis:

*H*_3_: Students’ multitasking behavior is more frequent, and their perceived learning performance is better.

### 2.4. The influence of education stage

Teenage students are known as digital natives, millennials, or i-Gen groups (Akçayır et al., 2016), which means that these students are taken for granted that they are good at using technology. However, although these teenage students were seen as a tech-savvy group, IT experience may have played a role in the differences. Multitasking behavior is malleable, and task-switching behavior can increase with usage (Cardoso-Leite et al., 2015; Moisala et al., 2016). Jeong et al. (2010) pointed out that college students have more media use time and exhibit more frequent media MBs than high school students. This shows that adolescent students may have differences in media MB across age groups due to different daily lifestyles. Given this, this study proposes the following hypothesis:

*H*_4_: The relationships among polychronicity (including time tangibility, involvement with people, and scheduling preference), multitasking behavior and perceived learning performance differ between high school students and college students.

## 3. Materials and methods

### 3.1. Participants

This cross-sectional study was administered among Taiwanese youths using convenience sampling methods. A total of 939 students from four high schools and two colleges responded to the questionnaire. After we excluded 66 incomplete and invalid questionnaires, 873 valid questionnaires remained. Furthermore, eight questionnaires were excluded because the participants self-reported that they had no experience using technology (such as mobile phones, computers or tablets) for learning. Ultimately, 865 questionnaires were included in the analysis. The background of the 865 respondents indicated that 51.21% were high school students and 48.79% were college students.

### 3.2. Instruments

#### 3.2.1. The scale of MB in online learning

This scale was mainly used to evaluate students’ participation in multiple tasks in the process of using digital technology, such as mobile phones and computers, to learn. These items were developed based upon related literature (Kaufman et al., 1991; Bluedorn et al., 1999; Poposki and Oswald, 2010; Haase et al., 2016). It is a 6-point Likert scale ranging from “never” (1) to “always” (6). The exploratory factor analysis (EFA) showed that the Kaiser–Meyer–Olkin (KMO) test value was 0.76 (*χ*^2^ = 1127.40, *p* < 0.001), the factor loadings ranged from 0.68 to 0.86, and the total explained variance was 62.49% (*α* = 0.80). The final scale contained 4 items (e.g., “When I use digital technology such as mobile phones and computers to learn, I do many things at the same time”) attributed to one factor.

#### 3.2.2. The scale of polychronicity in online learning

To measure the participants’ polychronicity in online learning, we used the Polychronicity in IT-Supported Learning Scale (Luo et al., 2021). The scale consisted of three subscales in the context of using digital technology such as mobile phones and computers to learn: time tangibility (TT), involvement with people (IP), and scheduling preference (SP). In their study, the subscales were reliable (*α* = 0.84 to 0.89), and the model fit of the exploratory factor analysis results was ideal (CMIN/DF = 1.84, RMSEA = 0.05, TLI = 0.97, CFI = 0.97, SRMR = 0.043). In this study, the reliability of each aspect of the scale is: TT (α = 0.90), IP (*α* = 0.87), and SP (*α* = 0.85).

#### 3.2.3. The scale of perceived online learning performance

This scale was mainly used to evaluate students’ perceptions of the effectiveness of online learning, and these items were developed based upon related literature (e.g., Hsia and Tseng, 2005; Liu and Wu, 2017). Responses were given on a 6-point Likert scale ranging from 1 (definitely disagree) to 6 (definitely agree). The EFA showed that the KMO test value was 0.92 (*χ*^2^ = 6384.59, *p <* 0.001), the factor loadings ranged from 0.71 to 0.84, and the total explained variance was 78.48%. The scale was divided into two aspects: knowledge acquisition (KA) contained 4 items (*α* = 0.92; e.g., “Online learning allows me to learn more knowledge”) and learning satisfaction (LS) contained 5 items (*α* = 0.92; e.g., “I am satisfied that online learning has improved my confidence in learning”).

### 3.3. Research ethics

All participation was voluntary, anonymous and confidential. We did not collect any information that could be provided to the participants, and the participants had the right to refuse to participate in the study at any time without any penalty. The analysis results are also presented in a holistic manner.

## 4. Results

### 4.1. Hypothetical SEM test

SEM for the total sample showed that $\chi_{\left(285\right)}^{2}=1351.84,p<0.001.$ The test failed to obtain nonsignificant results, likely because the $\chi^{2}$ value is sensitive to the number of cases (Bergh, 2015). Therefore, in large samples, the $\chi^{2}$ value may not be an appropriate indicator, and alternative indicators will be needed (Luo et al., 2021). Other indicators used in this study showed that *χ*^2^/*df* = 4.74, TLI = 0.92, CFI = 0.93, and RMSEA = 0.07, all satisfying the following criteria: *χ*^2^/*df*  <  5 (Jöreskog and Sörbom, 1993), TLI > 0.90 (Bentler and Bonett, 1980), CFI > 0.90 (Li, 2006), and RMSEA < 0.08 (McDonald and Ho, 2002), indicating that the fit between the model and the observed data was good.

### 4.2. Path relationships between multitasking, polychronicity, and perceived learning performance

**Figure 1:**
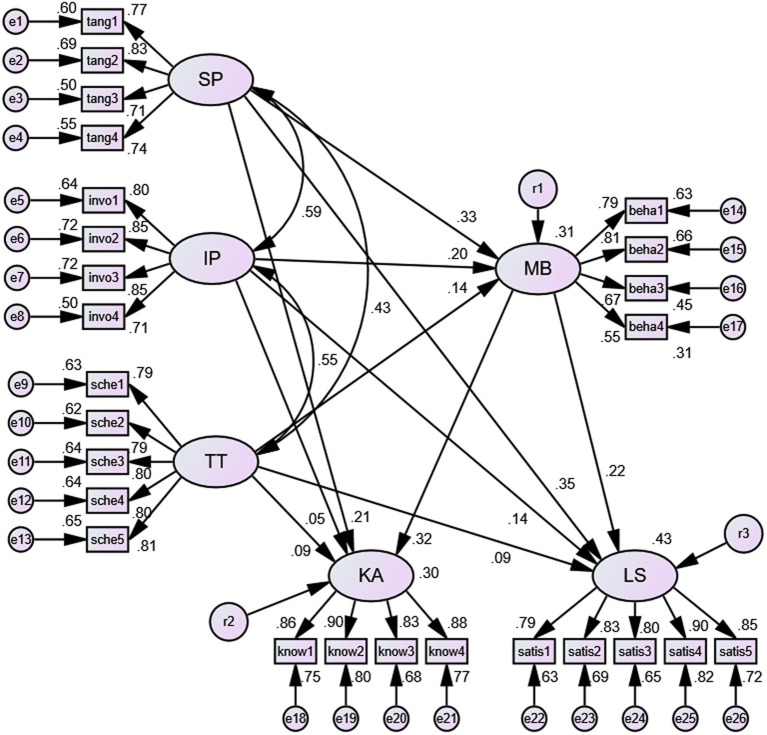
The standardization coefficient pattern of the SEM. SP = Scheduling preference, IP = Involvement with people, TT = Time tangibility, MB = Multitasking behavior, KA = Knowledge acquisition, LS = Learning satisfaction.

As shown in Figure 1 and Table 1, the results of the structural model assessment revealed that the 11 main paths in the whole sample are significant except the path of “involvement with people” to “knowledge acquisition.” Specifically, most of the research hypotheses H_1_ to H_3_ have gained statistical support.

**Table 1:** Path relationships among multitasking behavior, polychronicity, and perceived learning performance (*n* = 865).

Path	*β*	S.E	C.R.	*p*
*H_1_ Polychronicity to Multitasking behavior*				
Scheduling preference → Multitasking behavior	0.33^***^	0.05	6.68	<0.001
Involvement with people → Multitasking behavior	0.20^***^	0.05	3.91	<0.001
Time tangibility → Multitasking behavior	0.14^***^	0.04	3.19	<0.001
*H_2_ Polychronicity to perceived learning performance*				
Scheduling preference → Learning satisfaction	0.35^***^	0.05	7.81	<0.001
Scheduling preference → Knowledge acquisition	0.22^***^	0.05	4.53	<0.001
Involvement with people → Learning satisfaction	0.14^***^	0.05	3.21	<0.001
Involvement with people → Knowledge acquisition	0.05	0.05	1.00	0.32
Time tangibility → Learning satisfaction	0.10^*^	0.04	2.49	0.013
Time tangibility → Knowledge acquisition	0.09^*^	0.04	2.13	0.034
*H_3_ Multitasking behavior to perceived learning performance*				
Multitasking behavior → Learning satisfaction	0.22^***^	0.04	5.51	<0.001
Multitasking behavior → Knowledge acquisition	0.33^***^	0.04	7.37	<0.001

^*^p < 0.05, ^**^p < 0.01, ^***^p < 0.001.

### 4.3. Comparison the differences between education levels

The independent sample t test analysis results in Table 2 show no significant difference between the high school and college students in IP or KA. However, the college students scored significantly higher than the high school students in MB, TT, SP, and LS.

**Table 2:** T test of multitasking, polychronicity, and perceived learning performance across educational levels.

	High school students		College students		*t* Value	*p* Value
	Mean	SD	Mean	SD		
Multitasking behavior	3.61	1.02	3.87	0.91	−3.91^***^	<0.001
*Polychronicity*						
Time tangibility	3.71	1.16	3.85	0.98	−2.00^*^	0.045
Involvement with people	3.68	1.15	3.67	1.10	0.04	0.97
Scheduling preference	3.41	1.13	3.80	1.03	−5.40^***^	<0.001
*Perceived learning performance*						
Knowledge acquisition	4.54	0.99	4.43	1.00	1.58	0.11
Learning satisfaction	3.92	1.12	4.11	0.99	−2.60^**^	0.009

^*^p < 0.05, ^**^p < 0.01, ^***^p < 0.001.

The paths of the SEM were examined according to education levels. The goodness-of-fit indices for the high school student sample were $\chi_{\left(285\right)}^{2}=766.37,p<0.001,$ *χ*^2^/*df* = 2.69, TLI = 0.93, CFI = 0.93, and RMSEA = 0.06; the goodness-of-fit indices for the college student sample were $\chi_{\left(285\right)}^{2}=1057.83,p<0.001,$ *χ*^2^/*df* = 3.71, TLI = 0.89, CFI = 0.90, and RMSEA = 0.08. In general, the criteria were very close to or higher than the standard, indicating acceptable goodness-of-fit for the sample data and justifying further analysis.

Table 3 shows the estimates of the hypothetical SEM for the two subsamples. In the high school student sample, the path relationship between IP and KA was nonsignificant. In addition, SP had no significant effect on either LS or KA. In the college student sample, IP had no significant effect on MB, LS, or KA.

**Table 3:** Cross-group path coefficient invariance test results.

Path	Default model		Moderator model		Δ $\chi^{2}$	*p*	Path coefficient	
	$\chi^{2}$	*df*	$\chi^{2}$	*df*			High school	College
SP → MB	1824.22	570	1825.73	571	1.51	0.22	0.26^***^	0.37^***^
IP → MB	1824.22	570	1829.64	571	5.42	0.02	0.30^***^	0.08
TT → MB	1824.22	570	1824.40	571	0.18	0.67	0.14^*^	0.15^*^
SP → LS	1824.22	570	1824.89	571	0.67	0.41	0.36^***^	0.28^***^
SP → KA	1824.22	570	1824.23	571	0.002	0.96	0.25^***^	0.21^**^
IP → LS	1824.22	570	1825.41	571	1.19	0.28	0.17^*^	0.09
IP → KA	1824.22	570	1824.34	571	0.12	0.73	0.00	0.04
TT → LS	1824.22	570	1833.76	571	9.54	0.002	0.02	0.25^***^
TT → KA	1824.22	570	1827.08	571	2.85	0.09	0.05	0.17^*^
MB → LS	1824.22	570	1824.68	571	0.46	0.50	0.19^***^	0.26^***^
MB → KA	1824.22	570	1824.64	571	0.42	0.52	0.33^***^	0.34^***^

SP = Scheduling preference, IP = Involvement with people, TT = Time tangibility, MB = Multitasking behavior, KA = Knowledge acquisition, LS = Learning satisfaction.

^*^p < 0.05, ^**^p < 0.01, ^***^p < 0.001.

**Figure 2:**
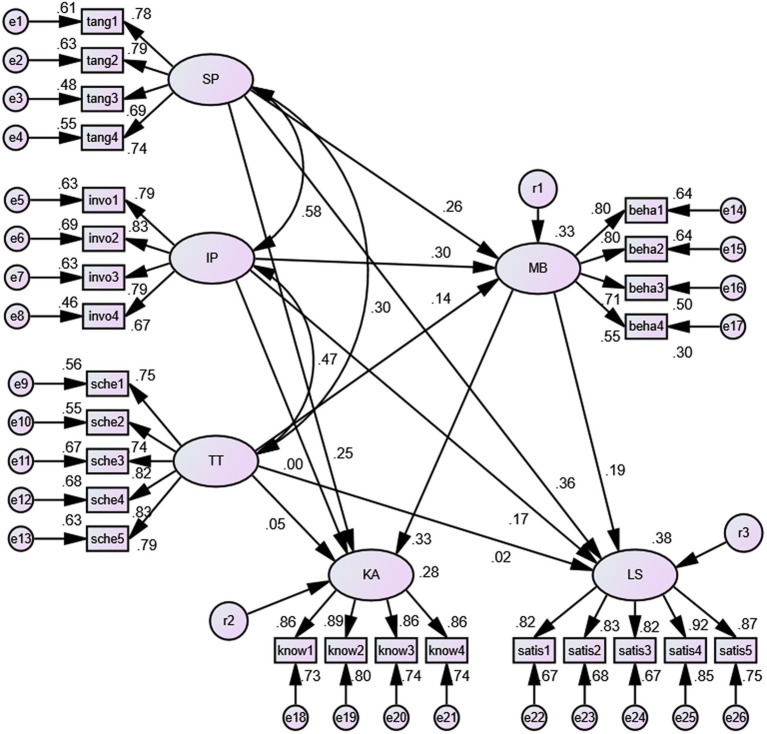
The standardization coefficient pattern of the SEM for the high school student sample. SP = Scheduling preference, IP = Involvement with people, TT = Time tangibility, MB = Multitasking behavior, KA = Knowledge acquisition, LS = Learning satisfaction.

**Figure 3:**
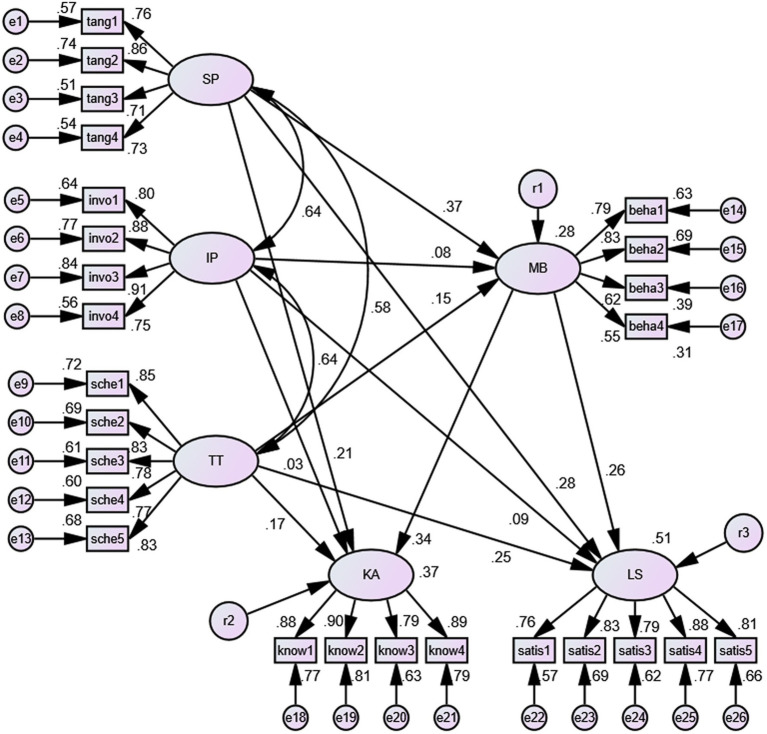
The standardization coefficient pattern of the SEM for the college student sample. SP = Scheduling preference, IP = Involvement with people, TT = Time tangibility, MB = Multitasking behavior, KA = Knowledge acquisition, LS = Learning satisfaction.

Furthermore, multigroup analysis was performed, which allowed analysis of the coefficient of the different paths of the two groups. As shown in Table 3, there were significant differences between the groups on the paths between IP to MB and SP to LS. The research hypotheses were partially supported for H_4_ (Figures 2, 3).

## 5. Discussion

In the predictive relationships of the entire sample, “scheduling preference” is an important predictor of “multitasking behavior” and perceived learning performance. This result is consistent with the general intuitive assumption that when learners prefer to do multiple things in a given time period and believe that this method is the best way to do things, they are more likely to adopt “multitasking behavior” (König et al., 2010); furthermore, a multitasking learning environment is in line with their personal interests and needs, which increases their satisfaction (Sanderson, 2012; Chuang et al., 2016). In addition, “time tangibility” can also predict “multitasking behavior” and perceived learning performance. That is, people who prefer to work in a timely, timesaving, and schedule-based manner actually participate in more tasks simultaneously and experience higher satisfaction in the process of using digital technology to learn. Luo et al. (2021) and Nonis et al. (2005) pointed out that “time tangibility,” which is closely related to time management, enables people to complete tasks on time or even deal with more tasks, and it is in line with the online learning environment that requires learners to perform learning tasks in a polychronic manner (Lee and Perry, 2001; Capdeferro et al., 2014; Lepp et al., 2019). Finally, the “involvement with people” aspect of polychronicity in online learning did not predict “knowledge acquisition.” It is possible that although emerging IT has multiple interpersonal interaction functions, Taiwanese students still seldom use these interactive functions in IT-supported learning, possibly because obedience and silence in classroom learning have long been aspects of the learning culture of Chinese students (Luo and Yang, 2018), which may extend to virtual online learning environments. Therefore, “involvement with people” may not reflect knowledge acquisition in the online learning environment.

Jeong et al. (2010) found that the frequency of media multitasking behaviors differed between high school students and college students because of the differences in their daily life schedules. This study aligns with a previous study (Jeong et al., 2010) and further finds that college students’ frequency of “multitasking behavior,” “scheduling preference” and “time tangibility” and their “learning satisfaction” in multitasking online learning environments were significantly higher than those of high school students. This may be because college students have more experience in using technology to learn than high school students (Jeong et al., 2010), which is evidenced in studies revealing more time overall in internet usage (Taiwan Network Information Center, 2018) and a higher percentage of internet addiction than high school students (Writer, 2022). Personal possession of technological media increases individuals’ use of technological media. Accessibility provides individuals with more opportunities for media MBs, thereby improving their self-efficacy in the use of technological media and increasing their preference for “multitasking behavior” in technological media (Srivastava et al., 2016).

Furthermore, this study found that, for students at different educational stages, different aspects of polychronicity had different predictive relationships on online learning “multitasking behavior” and perceived learning performance. “Scheduling preference” positively predicted “multitasking behavior” and perceived learning performance for both high school and college students. This result seems not surprising. As mentioned above, students who prefer and believe that multitasking is an ideal method are more likely to prefer multitasking behavior (König et al., 2010) and are more satisfied with the online learning environment, which may be potentially full of multitasking (Sanderson, 2012; Chuang et al., 2016). The statistical results of different education stages further explain that this phenomenon applies to many young people.

“Time tangibility” had a significant impact on the learning performance (including “knowledge acquisition” and “learning satisfaction”) of college students but not on that of high school students. Especially in “time tangibility” and “learning satisfaction,” the path coefficients of the two samples were significantly different. This may be because students with “time tangibility” feel that they can complete tasks on time or even complete more tasks through time management, resulting in higher academic performance (Nonis et al., 2005; Luo et al., 2021). In addition, “time tangibility,” which is related to time management, relates to students’ successful self-regulated learning. Liu et al. (2014) showed that time management was associated with self-regulated learning and that both positively predicted learning engagement. Wu (2017) found that students’ media multitasking indirectly predicted course grades through perceived attention and self-management strategies. Students’ learning performance on media multitasking can be improved by using attention regulation strategies, while students at higher stages of education have a clearer understanding of their learning patterns and are better able to regulate their learning in specific situations (Song and Vermunt, 2021).

High school students were more likely to be involved with people and had a higher frequency of multitasking behaviors, suggesting that media multitaskers were more likely to value online socializing (Zhong et al., 2011). However, this relationship did not apply to college students, possibly due to the aforementioned regulation strategies. College students can master multitasking more effectively than high school students through self-regulation strategies without being affected by interpersonal relationships. The positive effect of IP on “learning satisfaction” was also shown among high school students but not among college students, highlighting the importance of online socialization for high school students’ learning. For high school students who are primarily in adolescence, a desire to “fit in” with peers is characteristic (Wang and Chen, 2018), and “involvement with people” satisfies their desires, leading to positive “learning satisfaction.” However, “involvement with people” did not significantly predict “knowledge acquisition” in either college or high school students. Previous research has shown that interpersonal engagement in the classroom, such as collaborative learning and classroom dialog, must be a part of effective teacher scaffolding to produce good learning benefits (Muhonen et al., 2016; Chan, 2020). Therefore, online “involvement with people” may lack scaffolding guidance and effective collaboration and dialog, leading to reduced “knowledge acquisition.”

Interpretation of our findings should be made with caution due to the following limitations. First, due to individual subjectivity, students’ self-reports may not reflect their actual multitasking behaviors. Second, although we found that polychronicity is related to multitasking behavior and perceived learning performance, these variables are possibly not causally related in a practical sense. To clarify this issue, other research methods, such as experiments or observation methods, should be used in future research to deeply examine polychronicity, multitasking behavior and learning performance in an online learning environment. Finally, the “knowledge acquisition” investigated in this study is the result of students’ subjective perceptions, lacking objective data on academic knowledge and academic performance. Future research should consider collecting multiple academic achievements. Although there are limitations in this study, it is still helpful for developing a preliminary understanding of the polychronicity, multitasking and learning performance of online learning, as well as the differences across educational stages, and can be regarded as a basis for subsequent research development.

## 6. Conclusion

Media multitasking behavior has become a response to the application of digital technology, which also reflects that online learning multitasking behavior suitable for digital media ecology results in higher learning performance for both high school and college students. However, students’ mastery of self-regulation strategies at different educational stages caused by learning experiences may influence digital learning multitasking behavior and learning performance. In addition, the quality of involvement with people may be one of the antecedent factors affecting students’ online learning outcomes. Therefore, it is suggested that future research consider the moderating effects of self-regulation strategies and the quality of cooperative scaffolding and classroom dialog on polychronicity, multitasking behavior and learning performance in an online learning environment.

Future studies can also further examine what motivates heavy polychronic, or molychronic, learners to perform online multitasking behaviors in their learning engagement and how those learners activate and regulate cognitive capacity in their processing and consuming different media tasks at hand (cognitive/attentional control, executive functioning, media type attention, superficial/deep level of processing) and their cognitive/affective/physiological emotions (positive feelings accomplishments or negative stress) associated with their online multitasking and polychronicity activities (the perceived feeling of control, cognitive overload, perceived ability to process all information). Finally, future studies can further examine behavioral and neural as well as inhibiting or stimulating indicators related to the cognitive/affective/social mechanism of students’ engagement in polychronicity and multitasking behavior in an online learning environment.

## Data availability statement

The raw data supporting the conclusions of this article will be made available by the authors, without undue reservation.

## Ethics statement

Ethical review and approval was not required for the study on human participants in accordance with the local legislation and institutional requirements. The patients/participants provided their written informed consent to participate in this study.

## Author contributions

YL and SY contributed to conception and design of the study. CL collected the data and organized the database. YL performed the statistical analysis and wrote the first draft of the manuscript. YL, SK, SY, and CL wrote sections of the manuscript. All authors contributed to the article and approved the submitted version.

## Conflict of interest

The authors declare that the research was conducted in the absence of any commercial or financial relationships that could be construed as a potential conflict of interest.

## Publisher’s note

All claims expressed in this article are solely those of the authors and do not necessarily represent those of their affiliated organizations, or those of the publisher, the editors and the reviewers. Any product that may be evaluated in this article, or claim that may be made by its manufacturer, is not guaranteed or endorsed by the publisher.
